# Antidepressant prescriptions and associated factors in men with prostate cancer and their female partners

**DOI:** 10.1007/s11764-020-00947-y

**Published:** 2020-10-13

**Authors:** Tim J. Hartung, Ida Rask Moustsen, Signe Benzon Larsen, Elisabeth A. Wreford Andersen, Nis P. Suppli, Christoffer Johansen, Anne Tjønneland, Anne S. Friberg, Susanne K. Kjær, Klaus Brasso, Lars V. Kessing, Anja Mehnert, Susanne Oksbjerg Dalton

**Affiliations:** 1 Department of Medical Psychology and Medical Sociology, University Medical Center Leipzig, Philipp-Rosenthal-Strasse 55, 04103 Leipzig, Germany; 2 Unit of Survivorship, The Danish Cancer Society Research Center, Copenhagen, Denmark; 3 Copenhagen Prostate Cancer Center, Department of Urology, Rigshospitalet, Copenhagen University Hospital, Copenhagen, Denmark; 4 Statistics and Pharmacoepidemiology, The Danish Cancer Society Research Center, Copenhagen, Denmark; 5 Mental Health Centre Copenhagen, Gentofte Hospital, Copenhagen University Hospital, Copenhagen, Denmark; 6 Department of Oncology, Rigshospitalet, Copenhagen University Hospital, Copenhagen, Denmark; 7 Danish Cancer Society Research Center, Copenhagen, Denmark; 8 Department of Public Health, University of Copenhagen, Copenhagen, Denmark; 9 Unit of Virus, Lifestyle and Genes, The Danish Cancer Society Research Center, Copenhagen, Denmark; 10 Department of Gynecology, Rigshospitalet, Copenhagen University Hospital, Copenhagen, Denmark; 11 Psychiatric Center Copenhagen, Department O, Rigshospitalet, Copenhagen University Hospital, Copenhagen, Denmark; 12 Faculty of Health and Medical Sciences, University of Copenhagen, Copenhagen, Denmark

**Keywords:** Prostatic neoplasms, Depression, Anxiety disorders, Antidepressant agents, Diet, Life style

## Abstract

**Purpose:**

To estimate the risk of first-time antidepressant prescriptions as a proxy for depression or anxiety and associated risk factors in patients with prostate cancer and their female partners.

**Methods:**

We followed all men (*n* = 25,126) and their female cohabiting partners (*n* = 8785) without a history of cancer or antidepressants from the Danish Diet, Cancer and Health cohort from 1997 to 2014 or 2010, respectively. We estimated the cumulative incidence of first-time antidepressant prescriptions in men with prostate cancer compared with cancer-free men and their respective female partners, using the Danish National Prescription Registry. Sociodemographic, lifestyle-related, and clinical risk factors were assessed using Cox regression models.

**Results:**

A total of 1828 men were diagnosed with prostate cancer of whom 15% received antidepressants. The unadjusted hazard ratio of antidepressant prescription was 2.18 (95%CI, 1.92, 2.48) for men with prostate cancer and 1.27 (95%CI, 0.87, 1.85) for their partners, compared with cancer-free men and their partners, respectively. After adjusting for sociodemographic, lifestyle-related, and comorbidity factors, this risk was 2-fold to 4-fold increased among patients, but not significantly increased among partners. Significant risk factors among patients were curative and palliative treatment (vs. active surveillance and watchful waiting), nonlocalized disease, and short education.

**Conclusions:**

Men with prostate cancer have a higher risk of receiving antidepressant medication than cancer-free men. Clinical characteristics can help clinicians in identifying patients at a high risk of depression or anxiety.

**Implications for Cancer Survivors:**

Men with prostate cancer who experience symptoms of depression or anxiety should seek professional help early on. Patient education could aid in raising awareness and reducing the stigma associated with mental disorders.

**Electronic supplementary material:**

The online version of this article (10.1007/s11764-020-00947-y) contains supplementary material, which is available to authorized users.

## Introduction

Prostate cancer and its treatment can have a wide range of detrimental consequences for the patient. These may include fear of progression during active surveillance, erectile dysfunction, and urinary incontinence following curative therapy or decreased libido and mood disturbances following androgen deprivation therapy [1–4]. These problems have all been found to be associated with elevated psychosocial distress, anxiety, and depression [5].

When these issues disrupt the patients’ self-image, the relationship with their partner is often affected [3]. In addition, female partners of men with prostate cancer frequently report fear of what the future may hold, fear of recurrence or progression, and treatment-related concerns [6, 7]. Female partners tend to be more distressed than the patients themselves, and rates of major depression and generalized anxiety disorder may be up to twice as high as in the general population and remain elevated long after treatment [6–9]. However, most of the reported estimates stem from small studies with short follow-up. While there is some evidence that certain issues such as urinary incontinence may be particularly distressing for the partners, results on risk factors of mental disorders remain inconclusive [6].

Among men with prostate cancer, both depression and anxiety are reported more frequently than in the background population, leading to lower quality of life, reduced treatment adherence, and increased mortality [10–15]. However, it is unclear whether this elevated risk is truly a consequence of prostate cancer and its treatment or whether it is due to pre-cancer risk factors such as lifestyle or sociodemographic factors [16–18]. The available evidence on risk factors of mental disorders in men with prostate cancer comes from cross-sectional studies, which did not include lifestyle factors and do not allow for inferences about the direction of causation [5, 19].

Therefore, the aim of this longitudinal cohort study was to estimate the incidence of first redeemed prescriptions for antidepressants (FRPA) as an objective proxy of physician-diagnosed and -treated depression and anxiety disorders in both men with prostate cancer and in their female partners and to estimate the relative risk in these groups compared with the background populations. In addition, we aimed to identify risk factors of FRPA in patients with prostate cancer, including pre-cancer lifestyle, sociodemographic factors and comorbidity over time, as well as prostate cancer-specific clinical factors.

## Subjects and methods

### Participants

All persons living in the greater Copenhagen and Aarhus areas aged between 50 and 64 years without a previous history of cancer (*n* = 160,725) were invited to participate in the Diet, Cancer and Health cohort study [20]. The overall participation rate was 37% among women and 34% among men. Details about the study design, participation, and non-responder analyses have been published previously [20]. Baseline was defined as 1 January 1997. For participants who entered the study between January and May 1997, baseline was set to the date of study entry. Analyses within the group of patients with prostate cancer and the female partners of men with prostate cancer began at the date of prostate cancer diagnosis of the patient. All men and their female partners participating in this cohort were followed until 31 December 2014 (men) or 31 December 2010 (partners).

Study-specific exclusion criteria at baseline were (1) history of major psychiatric disorders, defined as at least one hospital contact for organic or substance-related mental disorders, schizophrenia, schizotypal or delusional disorder, bipolar or unipolar depression (ICD-8: 290 to 295.99, 296.19, 296.39, 303.00–304.99, and ICD-10: F00-F33); (2) history of cancer except non-melanoma skin cancer; and (3) one or more redeemed prescriptions for antidepressant medications (Anatomic Therapeutic Chemical classification system [ATC] code N06A, excluding Bupropion [N06AX12]).

### Measures

At enrollment, participants completed questionnaires concerning diet, lifestyle-, and health-related issues, including physical exercise (metabolic equivalents [MET] categorized in quartiles), smoking status (current, former, never), and alcohol consumption converted to gram/day (categorized into 0, 1–36, and > 36 g/day for men and 0, 1–24, and > 24 g/day for women, i.e., the recommended limit in Denmark at the time) [20]. A lab technician conducted anthropometrical measurements including height and weight. Body mass index (BMI) was categorized into underweight (< 18.5 kg/m^2^), normal weight (18.5–24.9 kg/m^2^), overweight (25–29.9 kg/m^2^), or obese (≥ 30 kg/m^2^).

In patients with prostate cancer, hospital records were screened to obtain levels of prostate-specific antigen (PSA) at the time of diagnosis, Gleason score, and treatment information (first-line treatment categorized as active surveillance, curative, palliative, and watchful waiting). Using the 2004 TNM classification, patients were categorized into localized (T1-2 N0,x M0,x and Tx N0 M0) vs. nonlocalized (all other TNM stages). As full information on cancer stage was not available in all hospital files, supplementary information was retrieved from the Danish Cancer Registry.

### Danish National Register Data

Unambiguous linkage of data from nationwide registers was secured by personal identification numbers. The National Cancer Registry [21], the National Prescription Registry [22], the National Patient Registry [23], the Danish Psychiatric Central Research Register [24], the Educational Register [25], and the Civil Registration System [26] were used for dates and ICD codes of cancer diagnoses and TNM stage where the information could not be obtained from hospital charts, dates of FRPA, Charlson Comorbidity Index (CCI), highest level of education (short, mandatory school only; medium, senior high school, or vocational education; long, higher education), cohabitation status (opposite-sex person with maximum of 15-year age difference, living in the same household), date of emigration, date of change in personal identification number, and date of death, respectively (see Supplementary Material S1 for more details).

### Statistical analysis

The outcome for all analyses was the first redeemed prescription for antidepressants (FRPA) following exposure (the male partner’s prostate cancer diagnosis). Antidepressant prescriptions have been successfully used as measure of physician-diagnosed depression and anxiety in patients with cancer [27]. In northern European countries, about two thirds of antidepressant prescriptions are issued to treat depression, followed by anxiety (20%), sleep disorders (10%), obsessive-compulsive disorder (< 5%), and neuropathic pain (< 5%) [28–30]. We used the date of the first rather than repeated prescriptions as it most closely corresponds to the point in time when a mental disorder requiring pharmacological treatment is diagnosed.

In all analyses of the male cohort, observations were censored by the following dates: diagnosis of non-prostate cancer and non-melanoma skin cancer (ICD-10 C-Diagnoses except C4A, C44, C61), hospital contacts for major psychiatric disorders not including depression (ICD-10 F00-F31), emigration, death or 31 December 2014. The same censoring criteria were applied in the partner cohort, with two additional dates: the date at which their partner was diagnosed with a non-prostate cancer and any change in cohabitation (including separation, divorce, or death of the male partner). As there were too few partners at risk after 2010, the end date for partners was set to 31 December 2010.

To estimate the unadjusted hazard ratios (HR) of FRPA for sociodemographic factors, lifestyle, and somatic comorbidity at baseline, we conducted univariate Cox regression analyses with age as the underlying time.

The adjusted HRs of FRPA were estimated in multivariable Cox regression models including education, BMI, MET, smoking status and alcohol consumption at enrollment, and CCI and cohabitation status (except for partners) as time-varying covariates, using age as the underlying time. The proportional hazard assumption was tested both graphically and using Schoenfeld residuals. Because the proportionality assumption was violated for prostate cancer diagnosis (prostate cancer vs. cancer-free) in the male cohort, we analyzed the interaction of age (cutoff 65 years) and having a prostate cancer diagnosis.

The incidence of FRPA after prostate cancer diagnosis in patients and their partners was estimated using cumulative incidence function (CIF) analyses. We statistically compared differences in cumulative incidence using Gray’s test [31].

To identify potential risk factors of FRPA in men with prostate cancer, we performed a multivariable Cox regression analysis using time since diagnosis as the underlying time, including the following independent variables: education, BMI, MET, smoking status and alcohol consumption at enrollment, CCI and cohabitation status as time-varying variables, tumor spread (localized vs. nonlocalized) at the time of diagnosis, and first-line treatment. The proportionality assumption was not violated. As a post hoc analysis, we compared patients in different treatment groups to cancer-free men, adjusting for the same covariates as in the first model.

All statistical analyses were performed in R version 3.5. [32] using the packages *prodlim* and *cmprsk* for cumulative incidence functions [33, 34], *survival* for Cox modeling [35], and *survminer* to test the proportional hazard assumption [36]. Cases with missing data were deleted list-wise before each analysis. Cohabitation status and education showed relevant amounts of missing data (4.1% and 2.0% missing values, respectively). All other variables had near-complete data with 0.4% or fewer missing values.

### Ethics approval and consent to participate

All participants provided written informed consent. The study was conducted in accordance with the Declaration of Helsinki and approved by the regional ethical committees on human studies in Copenhagen and Aarhus (File no.: (KF)11–037/01) and by the Danish Data Protection Agency (File no.: 2013-41-4232).

## Results

### Participants

**Fig. 1:**
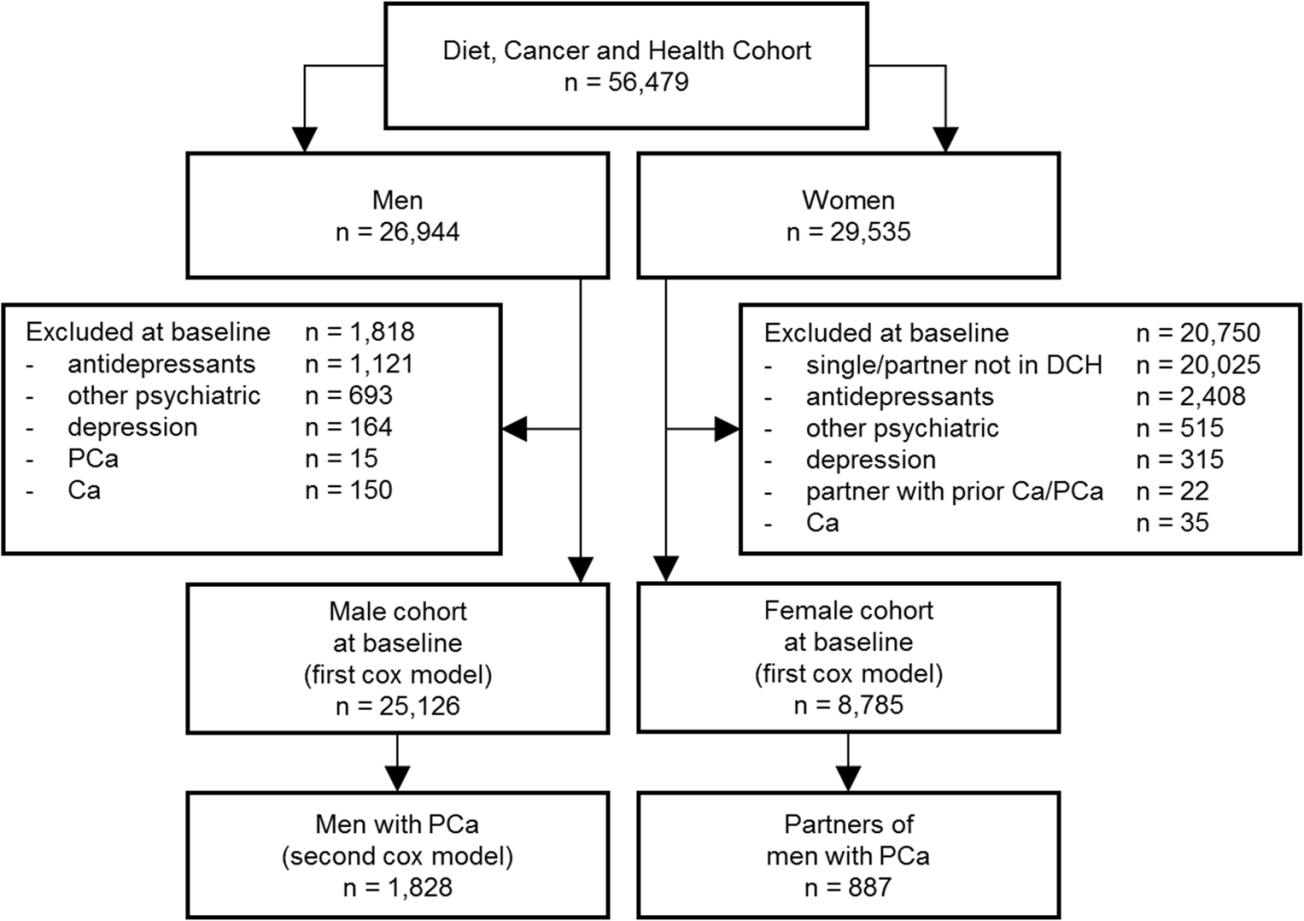
Participant flow for the analyses of first-time antidepressant prescriptions in men and their female partners participating in the prospective Danish Diet, Cancer and Health (DCH) cohort study. Abbreviations: PCa, prostate cancer; Ca, other malignant neoplasm

Out of 26,944 men in the Diet, Cancer and Health cohort, 25,126 men (93%) fulfilled the inclusion criteria (Fig. 1). At baseline, 8785 women from the Diet, Cancer and Health cohort who fulfilled the inclusion criteria were cohabiting with men from the cohort. Most men (median age 57 years; interquartile range 54 to 61 years) had medium education, were cohabiting, overweight, current or former smokers, drank moderate amounts of alcohol, and had no severe somatic comorbidities at baseline (Table 1). Female partners (median age 56 years; interquartile range 53 to 60 years) had similar patterns of education and somatic comorbidity, but their health behavior was more favorable than the men’s (Table 1). The median follow-up time was 17.8 years for the male cohort and 13.7 years for the female partner cohort.

**Table 1:** Baseline characteristics, person years at risk, and unadjusted hazard ratios of first-time antidepressant prescription events in male participants and their female partners in the Danish Diet, Cancer and Health cohort

		Male cohort				Female partners			
		Overall	PY	Events	HR (95%CI)	Overall	PY	Events	HR (95%CI)
Total		25,126	351,263	4330	-	8785	91,881	1315	-
Education	Short	3666 (1.9)	48,661	709	1	1706 (19.6)	17,146	298	1
	Medium	13,731 (55.8)	191,142	2486	0.91 (0.83 to 0.99)	5178 (59.4)	54,633	737	0.78 (0.68 to 0.89)
	Long	7228 (29.4)	106,263	1067	0.71 (0.64 to 0.78)	1836 (21.1)	19,807	267	0.78 (0.66 to 0.92)
Cohabiting	No	4492 (18.7)	60,245	906	1	-	-	-	-
	Yes	19,593 (81.3)	288,398	3345	0.77 (0.72 to 0.83)	8785 (100.0)	-	-	-
Body mass index	M (SD)	26.59 (3.58)	-	-	-	25.50 (4.13)	-	-	-
	Underweight	57 (0.2)	606	12	1.79 (1.02 to 3.16)	72 (0.8)	731	11	1.04 (0.57 to 1.89)
	Normal	8732 (34.8)	124,234	1381	1	4509 (51.4)	47,618	695	1
	Overweight	12,541 (49.9)	176,102	2235	1.14 (1.06 to 1.22)	3082 (35.1)	32,397	440	0.92 (0.82 to 1.04)
	Obese	3781 (15.1)	50,140	699	1.26 (1.15 to 1.38)	1116 (12.7)	11,356	169	1.02 (0.86 to 1.20)
Exercise level	M (SD)	33.01 (30.20)	-	-	-	32.45 (24.89)	-	-	-
	Top quartile	6257 (25.0)	87,349	1087	1	2168 (24.7)	22,898	289	1
	2nd quartile	6235 (24.9)	88,039	1032	0.95 (0.87 to 1.03)	2193 (25.0)	23,434	330	1.12 (0.96 to 1.31)
	3rd quartile	6130 (24.5)	86,431	1041	0.97 (0.90 to 1.06)	2209 (25.2)	23,099	370	1.28 (1.10 to 1.49)
	Bottom quart.	6430 (25.7)	88,499	1153	1.06 (0.97 to 1.15)	2201 (25.1)	22,614	320	1.13 (0.96 to 1.32)
Smoking	Never	6580 (26.2)	99,222	909	1	4358 (49.7)	47,564	583	1
	Former	8843 (35.3)	126,427	1476	1.24 (1.14 to 1.35)	2076 (23.7)	21,918	305	1.12 (0.97 to 1.28)
	Current	9662 (38.5)	125,112	1937	1.70 (1.58 to 1.84)	2342 (26.7)	22,625	424	1.54 (1.36 to 1.74)
Alcohol [g/day]	M (SD)	22.40 (20.18)	-	-	-	11.14 (10.49)	-	-	-
	0 g/day	876 (3.5)	11,292	192	1.48 (1.28 to 1.71)	791 (9.0)	7834	142	1.34 (1.13 to 1.60)
	≤ MRI	19,465 (77.8)	276,971	3230	1	7045 (80.5)	74,861	1003	1
	> MRI	4682 (18.7)	61,758	884	1.25 (1.16 to 1.35)	918 (10.5)	9195	162	1.32 (1.12 to 1.56)
Charlson comorbidity index	0	21,914 (87.2)	314,374	3536	1	8109 (92.3)	86,049	1149	1
	1	2477 (9.9)	29,729	583	1.70 (1.55 to 1.85)	562 (6.4)	5451	145	2.00 (1.68 to 2.37)
	≥ 2	735 (2.9)	7160	211	2.84 (2.45 to 3.29)	114 (1.3)	681	21	2.35 (1.52 to 3.61)

Overall values represent *n* (%) unless otherwise specified

*PY* person-years; events, first-time antidepressant prescriptions, *MRI* maximum recommended intake until 2010 (men: 21 Danish units/week or 36 g/day, women: 14 Danish units/week or 24 g/day)

### Sample characteristics and unadjusted associations with FRPA

Unadjusted Cox regression models showed that short education, living alone, smoking, high levels of alcohol consumption, and somatic comorbidity were associated with an overall higher risk of FRPA in both the overall male cohort and the partner cohort (Table 1). A BMI below or above normal weight was associated with a higher risk of FRPA in the male, but not the female cohort.

### Relative risk of FRPA

Patients with prostate cancer were followed for a median time of 4.3 years after receiving the diagnosis and female partners of patients for 3.1 years after their partner’s diagnosis (Table 2). Men with prostate cancer had a two-fold increased risk of FRPA and a two- to four-fold increased risk after adjusting for sociodemographic, lifestyle-related factors, and comorbidity, compared with cancer-free men (Table 3). Partners of men with prostate cancer had a non-significantly increased risk (Table 3).

**Table 2:** Medical characteristics of patients with prostate cancer (*n* = 1828)

Age	Median, IQR	69.5 [65.9, 73.1]
Year of diagnosis	1997–2001	191 (10%)
	2002–2006	486 (27%)
	2007–2011	774 (42%)
	2012–2014	377 (21%)
Tumor spread	Localized	1107 (64%)
	Nonlocalized	628 (36%)
	Missing	93 (5%)
TNM-T	T1	770 (42%)
	T2	402 (22%)
	T3	423 (23%)
	T4	3 (< 1%)
	Tx	230 (13%)
TNM-N	N0	428 (23%)
	N1-3	150 (8%)
	Nx	1250 (68%)
TNM-M	M0	849 (47%)
	M1	258 (14%)
	Mx	721 (39%)
Gleason score	Mean, SD	6.99 (1.17)
	< 7	528 (36%)
	7	521 (35%)
	> 7	432 (29%)
	Missing	347 (19%)
PSA	Median, IQR [ng/ml]	12.9 [7.4, 31.85]
	Missing	216 (12%)
Treatment	Active surveillance	127 (8%)
	Watchful waiting	237 (16%)
	Curative	666 (44%)
	Palliative	499 (33%)
	Missing	299 (16%)

Values represent *n* (valid %) at the time of diagnosis, unless otherwise specified

*IQR* interquartile range, *TNM* UICC tumor-lymph node metastasis stage, *PSA* prostate-specific antigen

**Table 3:** Unadjusted and adjusted hazard ratios for first-time antidepressant use in men with prostate cancer and their female partners compared with (partners of) cancer-free men

	Male cohort				Female partners			
			HR (95% CI)				HR (95% CI)	
	PY at risk	Events	Unadjusted	Adjusted	PY at risk	Events	Unadjusted	Adjusted
Cancer-free men	342,440	4057	1 (−)	1 (−)	90,733	1287	1 (−)	1 (−)
Men with PCa	8823	273	2.18 (1.92 to 2.48)	4.17 (2.99 to 5.82)^a^	1148	28	1.27 (0.87 to 1.85)	1.35 (0.92 to 1.97)
				1.97 (1.72 to 2.27)^b^				

^a^Age ≤ 65 years. ^b^Age > 65 years; adjusted models adjusted for education (short, medium, long), cohabitation status over time (yes/no), body mass index (underweight, normal, overweight, obese), daily exercise levels (metabolic equivalents), smoking (never, former, current), daily alcohol consumption (abstinent, below recommended maximum, above recommended daily maximum), Charlson comorbidity index over time (0, 1, ≥ 2)

*HR* hazard ratio, *CI* confidence interval

**Fig. 2:**
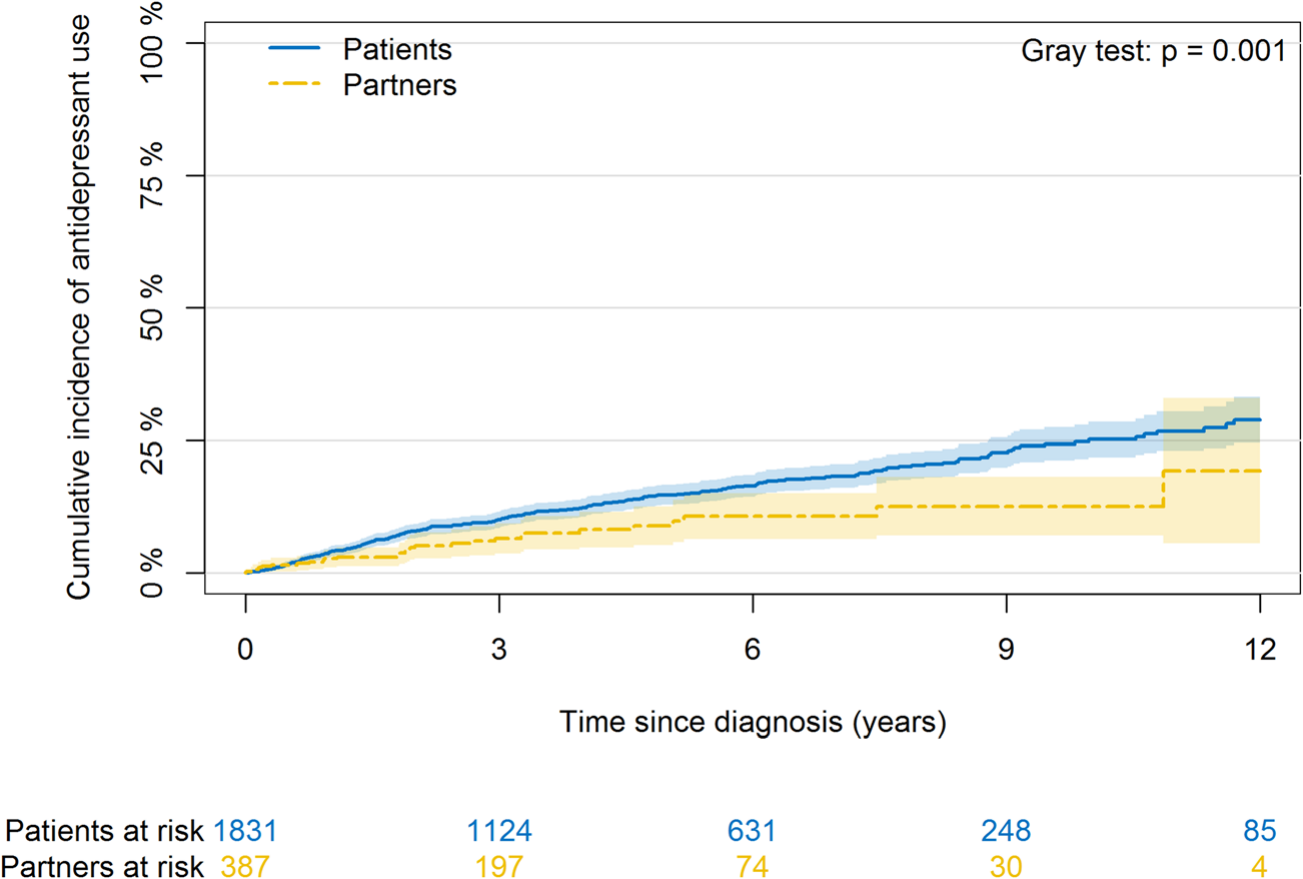
Cumulative incidence and 95% confidence interval of first-time antidepressant prescriptions among patients with prostate cancer and their female partners

After diagnosis with prostate cancer, patients had a significantly higher incidence of FRPA than their female partners (Gray test *p* = 0.001; Fig. 2).

### Sociodemographic and clinical characteristics of patients with prostate cancer

During the study period, 1828 men were diagnosed with prostate cancer (Fig. 1). At the time of diagnosis, patients with prostate cancer had a median age of 69 years (interquartile range, 66 to 73 years) and 85% were cohabiting with a partner. Patients’ medical characteristics are shown in Table 2. Patients assigned to palliative treatment received androgen deprivation therapy in more than 90% of the cases.

### Risk factors of FRPA in patients with prostate cancer

Palliative cancer treatment, curative treatment (compared with active surveillance/watchful waiting), non-localized disease, short education, and living alone were significantly associated with a higher HR of FRPA (Table 4). When active surveillance and watchful waiting were entered as separate categories into the analysis, both treatments showed a non-significant association with a lower risk of FRPA than patients in curative treatment (HR, 0.33; 95% CI, 0.10 to 1.05; and HR, 0.54; 95% CI, 0.29 to 1.03, respectively). An adjusted post hoc model showed that patients receiving active surveillance or watchful waiting had a similar risk as cancer-free men (HR 0.74, 95% CI, 0.48 to 1.14), while patients receiving curative (HR, 1.87; 95% CI, 1.52 to 2.30) or palliative treatment (HR 4.70; 95% CI, 3.90 to 5.66) had significantly higher risks of FRPA.

**Table 4:** Adjusted multivariable Cox regression model for factors associated with risk of first-time antidepressant prescription in 1828 men with prostate cancer

		PY	Events	HR (95% CI)
Education	Short	1171	58	1
	Medium	4580	139	0.59 (0.42 to 0.84)
	Long	2967	72	0.50 (0.34 to 0.74)
Cohabitation	No	1880	72	1
	Yes	6894	191	0.72 (0.53 to 0.98)
Body mass index	Normal	3187	89	1
	Overweight/obese	5603	184	1.04 (0.79 to 1.38)
Exercise level	Top quartile	2214	73	1
	2nd quartile	2178	66	0.85 (0.58 to 1.23)
	3rd quartile	2211	72	0.96 (0.67 to 1.37)
	Bottom quartile	2211	61	0.69 (0.47 to 1.01)
Smoking	Never	2774	70	1
	Former	3375	96	1.15 (0.82 to 1.63)
	Current	2664	106	1.32 (0.93 to 1.86)
Alcohol	0 units/week	309	8	0.77 (0.35 to 1.69)
	≤ MRI	7063	210	1
	> MRI	1419	53	1.00 (0.70 to 1.43)
Charlson comorbidity index	0	5885	160	1
	1	1793	65	1.23 (0.89 to 1.69)
	≥ 2	1145	48	1.14 (0.75 to 1.72)
Year of diagnosis	1997–2001	1437	56	0.57 (0.31 to 1.04)
	2002–2006	3339	96	0.61 (0.36 to 1.02)
	2007–2011	3531	98	0.59 (0.36 to 0.98)
	2012–2014	516	23	1
Tumor spread	Localized	5847	131	1
	Nonlocalized	1515	104	1.74 (1.26 to 2.40)
First-line treatment	AS/WW	1773	22	1
	Curative	4202	95	2.05 (1.24 to 3.41)
	Palliative	1828	128	4.00 (2.33 to 6.88)

Overall: 8823 PY, 273 events. Tumor spread was assessed at the time of diagnosis

*PY* person-years; events, first-time antidepressant prescriptions, *HR* hazard ratio, *MRI* maximum recommended intake until 2010 (men: 21 Danish units/week or 36 g/day), *AS* active surveillance, *WW* watchful waiting

### Sensitivity analyses

Sensitivity analyses adjusting for calendar period or date of diagnosis revealed no substantial effects on the HRs observed (data not shown).

## Discussion

In this large longitudinal study, men with prostate cancer had a two- to four-fold higher risk of being prescribed antidepressants for the first time (FRPA) compared with cancer-free men after adjusting for age, sociodemographic factors, lifestyle, and comorbidity. Partners of men with prostate cancer showed a non-significantly increased risk of FRPA compared with partners of cancer-free men (HR, 1.34; 95% CI, 0.92 to 1.96). We further identified three easily assessable risk factors of FRPA: curative/palliative treatment, nonlocalized tumor spread at the time of diagnosis, and short education.

Treatment type was the most important risk factor of FRPA in patients with prostate cancer. The observation that patients undergoing active surveillance had a lower risk of FRPA than patients in curative treatment is consistent with previous studies [4]. Data on watchful waiting are scarce, but in the SPCG-4 study, no difference in depression between patients receiving radical prostatectomy and watchful waiting was found [37].

Radical prostatectomy can lead to functional sequelae including urinary incontinence (prevalence up to 31% after surgery) and erectile dysfunction (up to 46%) [1, 2]. These sequelae have been found to be associated with depression, a relationship likely mediated by psychosocial factors such as relationship quality, social isolation, self-esteem, and negative cognitions [5, 38]. In turn, these relationships may be moderated by deeper-rooted beliefs about masculinity such as conformity to ideals of self-reliance, emotional control, dominance, and sexual performance [39, 40]. However, our results are in contrast to recent findings from the ProtecT trial which showed no difference in patient-reported depressive symptoms, anxiety, and mental quality of life between curative treatment and active surveillance [41].

On the one hand, any potentially lethal prognosis represents an existential threat and is associated with anticipated losses and grief; on the other hand, there may be prostate cancer-specific factors which explain the high risk of FRPA associated with palliative treatment in our cohort. Palliative treatment consisted in androgen deprivation therapy in more than 90% of cases. Androgen deprivation therapy may increase the risk of depression both directly and indirectly: first, many depressive symptoms may be a direct result of low testosterone levels [3, 42, 43]; second, androgen deprivation therapy has been shown to have negative effects on men’s self-image, sexual desire, erectile function, ability to become aroused, and to achieve orgasm, all of which impair sexual function and may upset sexual relationships [3]. This, in turn, is associated with an increased risk of depression.

Short education was the only significant cancer-independent risk factor of FRPA in patients with prostate cancer. This is consistent with the literature, as socio-economic position, which may be measured by educational level, has long been identified as a risk factor of depression in the general population [44]. However, cohabitation consistently showed a non-significant protective effect in all three models, which might have been significant with higher statistical power. The link between relationship status and depression is likely to be more complex than this and the risk of depression may particularly be associated with the dissolution of a relationship and strongly moderated by prior depression [45].

Lifestyle variables did not appear to be relevant risk factors of FRPA in patients with prostate cancer. While patients with a history of smoking showed a non-significant trend towards an increased risk of FRPA, alcohol intake and BMI showed no such association. Although a recent meta-analysis found convincing evidence that physical exercise is a protective factor against incident depression [46], we were not able to observe this effect. These findings may partly be due to the fact that men with cancer tend to change their health behavior after receiving the diagnosis [47].

The risk of FRPA was roughly constant over time after receiving a prostate cancer diagnosis, which corresponds to the roughly linear cumulative incidence function. However, fluctuations in psychosocial symptom burden may occur, e.g., an increase in anxiety before a surgery or in depression symptoms after experiencing treatment side effects, even if they do not lead to an immediate prescription of antidepressant medication.

Partners of men with prostate cancer did not show a statistically significantly increased risk of depression compared with partners of cancer-free men. This observation should be interpreted with caution. First, depression is more common in the female than in the male population. Therefore, the same absolute increase in risk corresponds to a smaller increase in relative risk. Second, due to the registry-based design, partners could not be followed after a change in cohabitation, including death of their cohabiting partner. It is plausible that women may face a higher risk of depression after change of cohabitation status, be it due to their partner’s death or for other reasons [48]. Third, the analysis of partners had a lower statistical power than in the male cohort (91,881 vs. 351,263 person-years overall). The observed 35% higher risk of FRPA may thus be replicable with statistical significance in a larger sample. In part, the discrepancy between patients and their partners may also be explained by surveillance bias: mental disorders may be more likely to be diagnosed and treated in patients than in their partners, because the former have more contact with health care professionals than the latter.

### Strengths and limitations

Strengths of our study include the prospective, longitudinal design, including detailed and objective data from before the cancer diagnosis until up to 18 years after the diagnosis and following both patients and their partners. The inclusion of near-complete data on lifestyle factors and detailed clinical data as well as the time-varying impact of cohabitation and somatic comorbidity further increase the scope of our analyses and the robustness of our results.

Our results ought to be interpreted in light of the following limitations: participation in the Diet, Cancer and Health cohort was associated with longer education and better overall health [20, 49]. Thus, it is likely that there is less variance in both general and mental health and our analyses may underestimate the actual effects. Lifestyle variables were self-reported, assessed only at enrollment, and may have changed over time [47]. Due to the registry-based assessment of cohabitation status based on sex, age, and address, it cannot be ruled out that some of the women identified as partners may actually be flat-mates or friends living in the same apartment. However, in this age group, such mislabeling is rare. Despite the large cohort, the power of the partner analyses was insufficient to confirm the significance of relatively weak associations. It should be noted that results on treatment refer to first-line treatment and some patients may have received other treatments later on. Antidepressants may be prescribed for issues other than depression and anxiety and a prescription thus does not exactly correspond to a diagnosis of these mental disorders. However, antidepressant prescriptions can be seen as a proxy for a psychological or neurological issue that was deemed as requiring pharmacological treatment by a physician and thus as an important group of intermediate and late effects of cancer and its treatment. As antidepressants are more frequently described for depression than for anxiety disorders, we did not use prior hospital stays due to anxiety as an exclusion criterion, which represents a small methodological asymmetry in the exclusion criteria.

## Conclusion

Our results suggest that men with prostate cancer face an increased risk of receiving a prescription for antidepressants, i.e., physician-treated depression and/or anxiety disorders. We identified three relevant risk factors of antidepressant treatment in men with prostate cancer (treatment regimen, tumor spread at the time of diagnosis, and education), which can aid clinicians in identifying patients at risk within the growing population of prostate cancer survivors. Partners of men with prostate cancer did not have a statistically significantly increased risk of antidepressant use compared with partners of cancer-free men, a finding which future research should attempt to replicate and investigate further. Despite its late onset and relatively good prognosis, prostate cancer can have substantial effects on mental health in both patients and their partners, especially in advanced phases of the disease.

## Electronic supplementary material

ESM 1 (DOCX 17 kb).

## Data Availability

The raw data of our study cannot be shared, as it contains register data on Statistics Denmark which does not permit data sharing.
