# Supplementary material for: Antidepressant prescriptions and associated factors in men with prostate cancer and their female partners
Source: J Cancer Surviv. 2020 Oct 13;15(4):536–45. doi: 10.1007/s11764-020-00947-y (PMC8272693; doi:10.1007/s11764-020-00947-y)
Supplement: Supplementary file 1 — (DOCX 17 kb). [file 11764_2020_947_MOESM1_ESM.docx]

**Supplementary Materials**

**Supplementary Material S1**: Detailed description of national register data used.

- The Cancer Registry holds accurate and nearly complete information on all cancer cases in Denmark since 1943 [1] and was used for dates and ICD codes of cancer diagnoses and prostate cancer TNM-stage (after 2003) and tumor spread (localized, regional, distant; until 2003) where the information could not be obtained from hospital charts.
- The National Prescription Registry holds complete and valid records of all prescriptions redeemed in Danish pharmacies since 1995, including those administered in nursing homes but excluding drugs directly supplied by hospitals [2]. Available data include drug name, date of redemption and ATC code and was used for dates of redeemed prescriptions of antidepressants.
- The National Patient Registry contains data for all in-patient hospital admissions since 1978 and outpatient and emergency contacts since 1995, including diagnosis [3] and was used for dates of hospital contacts and admission diagnoses of mental disorders and the diagnoses included in the Charlson Comorbidity Index (CCI).
- The Danish Psychiatric Central Research Register [4] ﻿contains information on every psychiatric admission since 1969 and was used for dates of hospital contacts and admission diagnoses of mental disorders.
- The Education Register [5] was used for data on the highest finished degree of education. Information on education was categorized into short (mandatory school only), medium (high school or vocational education) or long (higher education).
- The Civil Registration System [6] was used for cohabitation status (living with partner, living alone), date of emigration, date of change in personal identification number and date of death.

**REFERENCES**

1. Gjerstorff ML. The Danish Cancer Registry. *Scand J Public Health*. 2011;39(7 Suppl):42-45. doi:10.1177/1403494810393562.

2. Pottegård A, Schmidt SAJ, Wallach-Kildemoes H, Sørensen HT, Hallas J, Schmidt M. Data resource profile: The Danish national prescription registry. *Int J Epidemiol*. 2017;46(3):798. doi:10.1093/ije/dyw213.

3. Schmidt M, Schmidt SAJ, Sandegaard JL, Ehrenstein V, Pedersen L, Sørensen HT. The Danish National Patient Registry: a review of content, data quality, and research potential. *Clin Epidemiol*. 2015;7:449-490. doi:10.2147/CLEP.S91125.

4. Mors O, Perto GP, Mortensen PB. The Danish psychiatric central research register. Scand J Public Health. 2011;39:54–7.

5. Jensen VM, Rasmussen AW. Danish Education Registers. *Scand J Public Health*. 2011;39(7 Suppl):91-94. doi:10.1177/1403494810394715.

6. Helweg-Larsen K. The Danish Register of Causes of Death. *Scand J Public Health*. 2011;39(7 Suppl):26-29. doi:10.1177/1403494811399958.
